# Crohn’s disease: a population-based study of surgery in the age of biological therapy

**DOI:** 10.1007/s00384-021-03930-w

**Published:** 2021-04-19

**Authors:** Christian Stöss, Maximilian Berlet, Stefan Reischl, Ulrich Nitsche, Marie-Christin Weber, Helmut Friess, Dirk Wilhelm, Philipp-Alexander Neumann

**Affiliations:** Klinikum rechts der Isar, School of Medicine, Department of Surgery, Technical University of Munich, Ismaninger Str. 22, 81675 Munich, Germany

**Keywords:** Crohn’s disease, IBD, Surgery, Immunotherapeutics

## Abstract

**Purpose:**

Despite primary conservative therapy for Crohn’s disease, a considerable proportion of patients ultimately needs to undergo surgery. Presumably, due to the increased use of biologics, the number of surgeries might have decreased. This study aimed to delineate current case numbers and trends in surgery in the era of biological therapy for Crohn’s disease.

**Methods:**

Nationwide standardized hospital discharge data (diagnosis-related groups statistics) from 2010 to 2017 were used. All patients who were admitted as inpatient Crohn’s disease cases in Germany were included. Time-related development of admission numbers, rate of surgery, morbidity, and mortality of inpatient Crohn’s disease cases were analyzed.

**Results:**

A total number of 201,165 Crohn’s disease cases were included. Within the analyzed time period, the total number of hospital admissions increased by 10.6% (*n* = 23,301 vs. 26,069). While gender and age distribution remained comparable, patients with comorbidities such as stenosis formation (2010: 10.1%, 2017: 13.4%) or malnutrition (2010: 0.8%, 2017: 3.2%) were increasingly admitted. The total number of all analyzed operations for Crohn’s disease increased by 7.5% (2010: *n* = 1567; 2017: *n* = 1694). On average, 6.8 ± 0.2% of all inpatient patients received ileocolonic resections. Procedures have increasingly been performed minimally invasive (2010: *n* = 353; 2017: *n* = 687). The number of postoperative complications remained low.

**Conclusion:**

Despite the development of novel immunotherapeutics, the number of patients requiring surgery for Crohn’s disease remains stable. Interestingly, patients have been increasingly hospitalized with stenosis and malnutrition. The trend towards more minimally invasive operations has not relevantly changed the rate of overall complications.

**Supplementary Information:**

The online version contains supplementary material available at 10.1007/s00384-021-03930-w.

## Introduction

The central role of medical treatment for Crohn’s disease (CD) is the early prevention of disease progression through immunosuppressive therapy. In recent decades, the development of biologic agents, such as antibodies against tumor necrosis factor (TNF) alpha, has led to significant change in the medical treatment of CD [1]. After the initial approval of the anti-TNF antagonist infliximab, medical therapy for CD has advanced rapidly. Recently, there has been a paradigm shift in the way medical treatment is approached: early use of biologics is increasingly performed as a “top-down-therapy” or at least “rapid step-up therapy” compared to the more traditional “step-up” approaches [2]. The search for additional treatment strategies has led to the development of a plethora of novel therapeutics in the field. It is speculated that with the steady increase in medical options, the number of operations in CD patients will diminish. However, treatment failure with TNF antagonists is observed in up to 30–50% of patients [2]. For the new drug targets, the reported efficacy of all drugs vs. placebo is still fairly limited [3]. Therefore, despite all medical advances, the introduction of biologics does not appear to have a major impact on the likelihood that a patient will or will not undergo surgery. Surgery is still required in a significant proportion of patients and constitutes an important part of the treatment algorithm in the management of CD [4].

The aim of this study was to analyze the development of CD with special reference to surgical treatment within the last decade. A population-based systematic analysis was performed to assess all inpatient CD cases from 2010 to 2017 in Germany. The number of hospitalizations and surgical procedures for CD were summarized, and surgical complications as well as in-hospital mortality are presented.

## Materials and methods

### Study design and setting

The present study is based on the national reimbursement system (G-DRG), which comprises inpatient data from all acute care hospitals in Germany. The national diagnosis-related groups (DRG) statistics contain all data records of inpatient treatments billed according to the DRG system in Germany. The information available for each of these inpatient cases include age, sex, diagnoses (coded according to the International Statistical Classification of Diseases and Related Health Problems, 10th revision, German modification, ICD-10-GM), procedures (coded according to the German Procedure Classification, OPS), length of hospital stay, and mode of discharge. The individual inpatient data of the DRG statistics of the years 2010 to 2017 were accessed remotely via the Research Data Centre of the Federal Statistical Office by means of controlled data processing [5]. The evaluation of the secondary data obtained for the present investigation does not require an ethics committee vote or support by the competent authority [6].

### Inclusion criteria and outcome variables

The units of analysis were inpatient cases who were admitted for Crohn’s disease. These cases were identified by a principal diagnosis of Crohn’s disease (K50.-). Main localization of CD was differentiated by using K50.0 (main manifestation of small intestine), K50.1 (main manifestation of large intestine), as well as K50.8- (upper GI plus combined manifestations in the upper and lower GI tract) and K50.9 for “Crohn’s disease, unspecified” (e.g., regional enteritis).

For analysis of the development of surgical procedures over time, the respective OPS codes of ileocecal resection and right hemicolectomy were chosen as sentinel procedures and defined as ileocolonic resections. For outcome comparison between laparoscopic and open procedures, numbers of the respective groups were pooled to be analyzed as either laparoscopic or open ileocecal resection or hemicolectomy, respectively. Clinical outcomes were assessed by in-hospital mortality, defined as death before discharge as well as secondary diagnoses associated with the respective procedure. To assess the quality of surgical procedures, standardized secondary diagnosis codes of the main postoperative complications were analyzed as follows: postoperative obstruction, surgical site infection (SSI), and anastomotic leakage. All definitions of diagnosis and procedure codes are displayed in Supplemental Table 1 (published electronically).

### Statistical methods

This study is following the “Strengthening the Reporting of Observational Studies in Epidemiology” (STROBE) Statement checklist [7]. All calculations were performed using SAS Enterprise Guide 7.13 (SAS Institute Inc., Cary, NC, USA). For graphical visualization, GraphPad Prism Version 9.0.0 (GraphPad Software, CA, USA) was used. Data were analyzed descriptively for every year of observation and are expressed as absolute and relative frequencies. Continuous variables are presented as mean with single standard deviation. Pearson’s chi-squared test or Mann-Whitney *U* test was used for statistical analyses where appropriate; *p* values < 0.05 were considered statistically significant (*p* < 0.05 = *; *p* < 0.01 = **; *p* < 0.001 = ***).

## Results

### Baseline characteristics

**Fig. 1:**
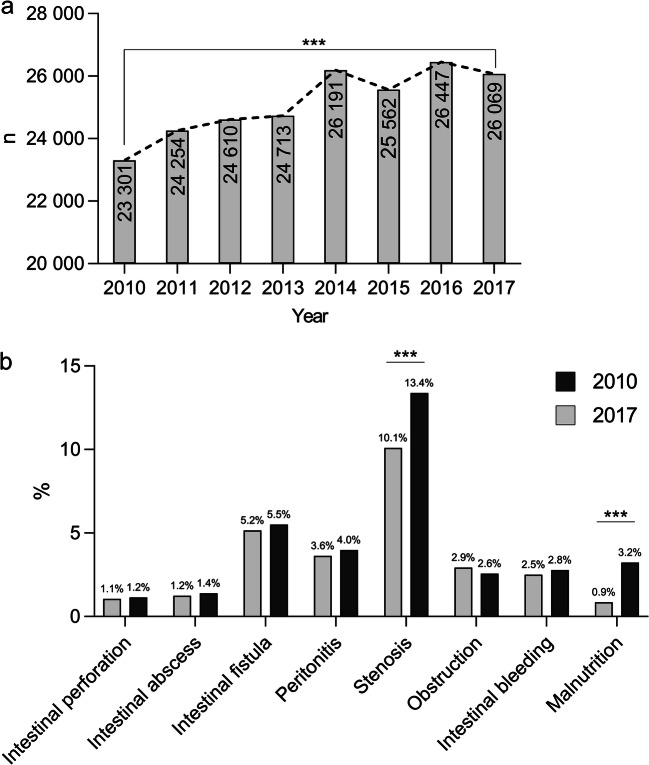
Development of inpatient cases with Crohn’s disease in Germany. **a** The total number of inpatient cases in Germany increased significantly over the study period. **b** Secondary diagnoses in 2010 compared to 2017 (relative numbers per year) with a significant increase of relative numbers for stenosis and malnutrition (*p* < 0.05 = *; *p* < 0.01 = **; *p* < 0.001 = ***)

A total of 201,165 inpatient cases of Crohn’s disease were counted within the analyzed time period. While the gender and age distribution for the disease did not change during the study period, the number of inpatient CD cases significantly increased by 10.6% (*n* = 23,301 in 2010 vs. *n* = 26,069 in 2017; *p* = 0.0005) (Fig. 1a). Emergency admissions accounted for 38% of all cases in 2010 compared to 45% in 2017. Coding for small intestine CD increased over the study period (2010: 34%; 2017: 38%), whereas CD of the large intestine remained widely the same (2010: 25%; 2017: 24%) (Table 1).

**Table 1:** Characteristics of inpatient Crohn’s disease cases

Total number of inpatient cases		2010		2011		2012		2013		2014		2015		2016		2017	
		*n*	%	*n*	%	*n*	%	*n*	%	*n*	%	*n*	%	*n*	%	*n*	%
		23,301	100	24,254	100	24,610	100	24,731	100	26,191	100	25,562	100	26,447	100	26,069	100
Age groups	< 15	1669	7	1744	7	1814^*^	7	1905	8	1937	7	1813	7	1873	7	1835^**^	7
	15–35	9323	40	9659	40	9794	40	10,146	41	10,663	41	10,314	40	10,645	40	10,328	40
	> 35	12,309	53	12,851	53	12,928	53	12,680	51	13,591	52	13,435	53	13,929	53	13,793	53
Gender	Female	13,100	56	13,636	56	13,642	56	13,572	55	14,553	56	14,103	55	14,502	55	14,015	54
Localization	Small intestine	7827	34	8241	34	8640	35	8850	36	9403	36	9224	36	9666	37	9912	38
	Large intestine	5874	25	6303	26	6025	25	5790	23	6000	23	5901	23	6261	24	6315	24
	Combined and upper GI tract^1^	5089	22	5555	23	5658	23	5783	23	6294	24	6274	25	6388	24	6112	23
	Crohn’s disease, unspecified^2^	4511	19	4155	17	4287	17	4308	17	4494	17	4163	16	4132	16	3730	14
Secondary diagnosis	Intestinal perforation	246	1.1	250	1.0	279	1.1	268	1.1	285	1.1	272	1.1	280	1.1	300	1.2
	Intraabdominal abscess	290	1.2	289	1.2	290	1.2	332	1.3	319	1.2	336	1.3	358	1.4	363	1.4
	Intestinal fistula	1202	5.2	1256	5.2	1275	5.2	1265	5.1	1307	5.0	1297	5.1	1471	5.6	1433	5.5
	Peritonitis	846	3.6	921	3.8	868	3.5	936	3.8	1001	3.8	948	3.7	982	3.7	1037	4.0
	Stenosis or stricture of the intestine	2351	10.1	2478	10.2	2344	9.5	2552	10.3	2942	11.2	3047	11.9	3317	12.5	3487	13.4
	Intestinal obstruction	682	2.9	721	3.0	688	2.8	717	2.9	780	3.0	663	2.6	761	2.9	669	2.6
	Intestinal bleeding	583	2.5	564	2.3	585	2.4	637	2.6	631	2.4	710	2.8	734	2.8	724	2.8
Malnutrition	Mild	54	0.2	60	0.2	76	0.3	143	0.6	172	0.7	92	0.4	177	0.7	192	0.7
	Moderate	77	0.3	108	0.4	124	0.5	210	0.9	238	0.9	228	0.9	274	1.0	294	1.1
	Severe	66	0.3	75	0.3	110	0.4	181	0.7	259	1.0	272	1.1	355	1.3	355	1.4
Emergency admission		8739	38	9451	39	9809	40	10,358	42	11,230	43	11,354	44	11,914	45	11,756	45

^*^Data was censored for the ICD code K50.1 (*n* = 74)

^**^Data was censored for the ICD codes K50.1 and K50.8 (*n* = 113)

^1^Includes a combined manifestation of the upper GI tract and several sections of the small and large intestine

^2^E.g., regional enteritis

### Crohn’s disease related secondary diagnoses

The main secondary diagnoses during the patients’ hospitalization were analyzed to assess disease severity. In particular, the incidence of intestinal stenosis, intestinal fistula formation, and peritonitis were analyzed. Overall, most of the secondary diagnoses were on a constant level during the analyzed time period: the rate of intestinal fistula formation was 5.2% in 2010 and 5.5% in 2017, abscess formation accounted for 1.2% in 2010 and 1.4% in 2017, and intestinal perforation for 1.1% in 2010 and 1.2% in 2017, respectively. The codes for intestinal obstruction/ileus (2010: 2.9%; 2017: 2.6%) or intestinal bleeding (2010: 2.5%; 2017: 2.8%) remained unchanged, such as the secondary diagnosis peritonitis (3.6% in 2010 and 4.0% in 2017). Interestingly, a significant increase for the secondary diagnosis of stenosis formation was observed (*p* < 0.001): in 2010, 2351 patients (10.1%) were hospitalized with stenosis, while in 2017, 3487 patients were admitted for CD and stenosis formation (13.4%). Analysis of the nutritional status of the patients revealed that, in 2010, 54 patients (0.2%) were mildly malnourished, 77 patients (0.3%) were diagnosed with moderate malnutrition, and 66 patients (0.3%) suffered of severe malnutrition. In 2017, 192 patients (0.7%) were mildly malnourished, 294 patients (1.1%) were diagnosed with moderate malnutrition, and 355 patients (1.4%) suffered from severe malnutrition. Taken together, the increase of malnourished patients over time was significant (*p* < 0.001). See Fig. 1b and Table 1 for all secondary diagnoses of CD.

### Surgical procedures for Crohn’s disease

**Fig. 2:**
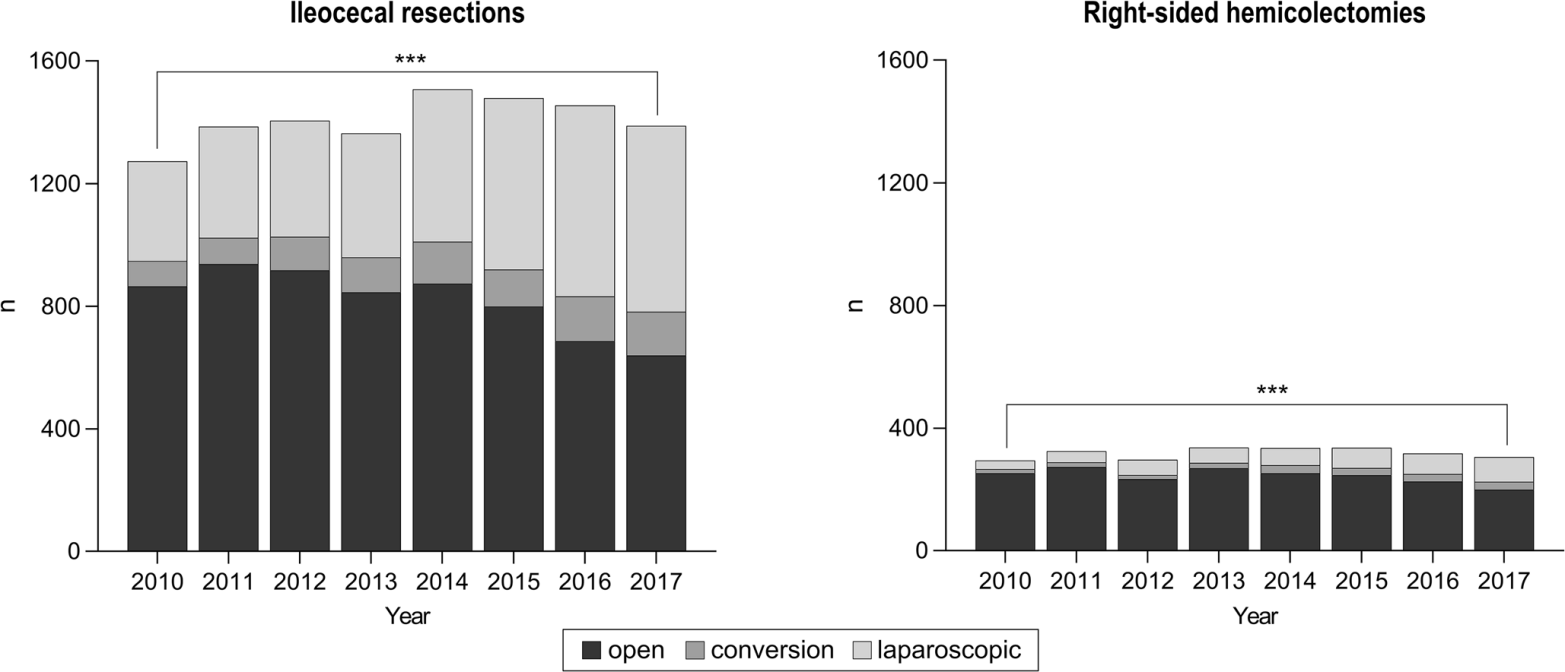
Development of ileocolonic resections for Crohn’s disease from 2010 to 2017. Absolute numbers of ileocecal resections (left) and right-sided hemicolectomies (right) for Crohn’s disease from 2010 to 2017 are depicted. The surgical approach (open vs. laparoscopic) has changed significantly over time (*p* < 0.05 = *; *p* < 0.01 = **; *p* < 0.001 = ***)

Since ileocolonic resections (ileocecal resection and segmental right-sided hemicolectomy) are the most common surgical procedures in CD, they were used to assess the surgical development in CD (Fig. 2). From 2010 to 2017, an average of 1726 ± 86 (6.8 ± 0.2%) ileocolonic operations (open and laparoscopically) were performed yearly. In 2010, a total number of 1567 ileocolonic operations (ileocecal resections: 1273; right-sided hemicolectomies: 294) were counted compared to a total of 1694 in 2017 (ileocecal resections: 1389; right-sided hemicolectomies: 305), which corresponds to a significant increase of 7.5% (*p* < 0.001). However, in relation to the increase in CD cases, the relative rate of ileocolonic resections remains at a constant level during the study period. Open ileocolonic procedures accounted for 1118 cases (71%; ileocecal resections: 55%; right-sided hemicolectomies: 16%) in 2010 compared to 838 cases (49%; ileocecal resections: 38%; right-sided hemicolectomies: 12%) in 2017. In contrast, laparoscopically performed operations increased from 353 (23%; ileocecal resections: 21%; right-sided hemicolectomies: 2%) to 687 (41%; ileocecal resections: 36%; right-sided hemicolectomies: 5%).

### Postoperative complications of ileocolonic resections in CD patients

**Fig. 3:**
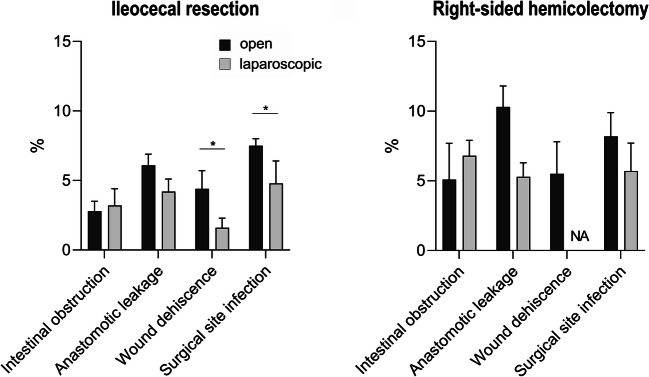
Postoperative complications following surgery for Crohn’s disease. Intestinal obstruction, anastomotic leakage, wound dehiscence, and surgical site infection are shown as postoperative complications following ileocecal resection or right-sided hemicolectomy. Laparoscopic ileocecal resection was associated with a lower rate of wound dehiscence and surgical site infection compared to an open approach. Postoperative complication rates tended to be higher with right-sided hemicolectomy (*p* < 0.05 = *; *p* < 0.01 = **; *p* < 0.001 = ***), NA, no data available

Postoperative morbidity and mortality in open and laparoscopic ileocolonic resections were studied to compare surgical outcomes following the respective techniques (Fig. 3).

For ileocecal procedures, the rate of postoperative ileus following an open approach was 2.8 ± 0.7% vs. 3.2 ± 1.2% in laparoscopic resections (*p* = 0.38). Anastomotic leakage (6.1 ± 0.8% vs. 4.2 ± 0.9; *p* = NA), postoperative wound dehiscence (4.4 ± 1.3% vs. 1.6 ± 0.7%; *p* = 0.03), or SSI (7.5 ± 0.5% vs. 4.8 ± 1.6; *p* = 0.03) were more common in open resections compared to laparoscopic procedures. Compared with ileocecal resections, the postoperative complication rates of right hemicolectomy were slightly higher regarding all variables: postoperative obstruction (open vs. lap; 5.1 ± 2.6% vs. 6.8 ± 1.1%; *p* = 0.88), anastomotic leakage (10.3 ± 1.5 vs. 5.3 ± 2.3%, *p* = NA), wound dehiscence (5.5 ± 2.3% vs. NA), and SSI (8.2 ± 1.7 vs. 5.7 ± 2.0%; *p* = 0.37).

In-hospital mortality was calculated at 0.6 ± 0.2% if a patient was admitted with CD and received ileocolonic resection. Interestingly, in-hospital mortality was 0.8 ± 0.3% when open ileocecal resection was performed and 2.1 ± 0.4% in the case of open hemicolectomy (*p* = 0.0015). For laparoscopic procedures, only complete data were available for laparoscopic hemicolectomy and indicated that no patient died during the whole period studied.

**Fig. 4:**
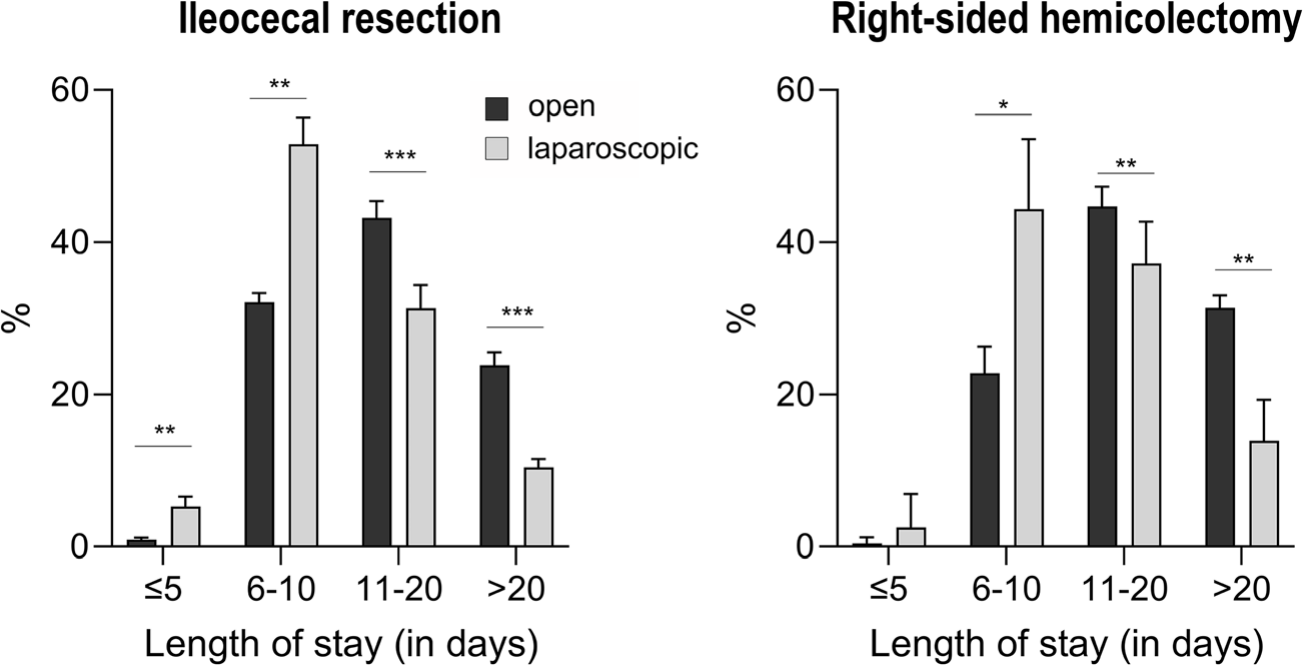
Postoperative length of stay. Distribution of cases with ileocecal resection or hemicolectomy relative to the length of stay. Cases with laparoscopic procedures had a significantly shorter length of stay. Additionally, cases with ileocecal resection tended to have shorter length of stay than cases with right-sided hemicolectomy (*p* < 0.05 = *; *p* < 0.01 = **; *p* < 0.001 = ***). %, proportion of overall cases

Regarding length of stay, it is notable that the cohort of patients who underwent laparoscopic ileocecal resection had a shorter length of stay compared with the open approach (length of stay, open vs. laparoscopic: ≤ 5 days: 0.9 vs. 5.3%; 6–10 days: 32.1 vs. 56.4%; 11–20 days: 42.7 vs. 29.0%; > 20 days: 23.2 vs. 8.8%). Overall, a higher proportion of hemicolectomy cases had a longer hospital stay independent of the approach (open vs. laparoscopic: ≤ 5 days: 0.4 vs. 2.5%; 6–10 days: 22.8 vs. 44.4%; 11–20 days: 44.7 vs. 37.2%; > 20 days: 31.4 vs. 13.9%) (Fig. 4).

## Discussion

More than 20 years after the introduction of biological therapy, an increasing variety of biological therapeutics are now available to treat patients with CD [8]. Nevertheless, surgery continues to play a significant role and a percentage of patients still require surgery for refractory disease or disease-related complications.

This analysis of more than 200,000 cases provides an overview of the absolute number of inpatient Crohn’s disease cases and surgical procedures in a Western world country from 2010 to 2017, revealing an increase in inpatient cases of almost 10%. Interestingly, CD-related complications such as intestinal stenosis or malnutrition were increasingly diagnosed. The relative rate of surgical interventions did not change during the analyzed time period. Hence, on average, about 6.8% of all Crohn’s disease inpatient cases received either an ileocecal resection or right-sided hemicolectomy. While the number of laparoscopic procedures increased, postoperative morbidity remained comparably low. Mortality was consistently low at 0.3%. Compared with open procedures, patients undergoing laparoscopic procedures had lower postoperative morbidity and shorter length of stay.

Previous studies suggest that recent advances in biological therapy for CD have led to a reduction in the number of surgeries for CD. For instance, Mao et al. recently reported a reduction in hospitalization and surgery due to the use of TNF antagonists [9]. In their systematic review and meta-analysis, they concluded that anti-TNF biologics reduced the odds of hospitalization by half and surgery by 33–77%. As their study focused on the use of biologicals within the setting of clinical trials, the situation in the real world is more heterogeneous, making it difficult to draw a conclusion about the specific effect of introducing TNF antagonists [10]. Another meta-analysis of population-based studies from 2013 showed a decline of surgeries for CD [11]. Nonetheless, subsequent studies have shown that this decline is not necessarily related to immunomodulators or biologicals, and progression to complicated disease was still common [12]. In a prospective population-based cohort study from 2000 to 2012, Golovics et al. showed that hospitalization and re-hospitalization rates at 1, 3, and 5 years of follow-up were still 32.3%, 45.5%, 53.7% and 13.6%, 23.9%, 29.8%, respectively. Hospitalizations in the first year were related to diagnostic procedures (37%), surgery (27%), or disease activity (21%) [13]. A Polish study based on National Health Insurance Fund data with 1393 patients from 2012 to 2014 reported a reduction in hospitalization and the need for surgery depending on previous treatments [14]. The latter is an important point to elaborate on. A population-based analysis from Manitoba, Canada, including 3400 patients, reported that only a small percentage of patients were being treated with infliximab in the first decade of the century [15], while a report from France from 2000 to 2008 demonstrated an overall use of 65% [16]. In this cohort, of all patients who required surgery, about 60% were treated with at least one biological. Altogether, the rate of biologic therapy varies in different populations, which might also be a confounding factor in our analysis.

In regard to CD-related comorbidities, a considerable increase in the frequency of stenosis formation was observed, which may indicate a tendency toward chronicity of disease rather than improvement in disease progression in the era of biological therapy. Immunosuppression might reduce intestinal inflammation but ultimately leads to fibrosis and obstructive courses of disease due to stenosis formation. In our analysis, this is accompanied by an increase in malnutrition, which could also point to a trend toward more chronic forms of disease.

Not only has medical therapy evolved, but surgical techniques have also advanced significantly over the past few years. With the advent of minimally invasive techniques and improvements in surgical equipment, surgical management of patients with CD can now be performed with less morbidity. With respect to surgical techniques, it is noticeable that laparoscopic procedures increased in 2017 compared with 2010 and accounted for nearly 50% of procedures in the later phase of our analysis. This applies to both ileocecal resection and right hemicolectomy.

As for postoperative complications in general, we did not observe a profound increase. Relative rates were consistently low, underlining the safety and efficacy of surgery in CD. Similar findings were recently presented by Mege et al. who demonstrated that despite a higher incidence of comorbidities, postoperative morbidity remained widely unchanged [17]. In addition, the authors reported an increase in laparoscopic procedures, which is comparable to our results. Here, our analysis revealed that overall postoperative complications remained at a low level. Further, coding of postoperative complications did occur less frequently following minimally invasive procedures. The latter might lead to the assumption that more complicated cases are still performed as open procedures. Nonetheless, our data show that surgery in CD patients is feasible to perform and safe even in the age of biological therapy.

The present study has several limitations that need to be addressed. First, cases with outpatient treatment and application of immunotherapeutics were not analyzed. Therefore, the overall development of CD cases in Germany is underestimated in our analysis. In addition, approximately 40% of all cases were admitted to the hospital as an emergency, which might have influenced the data on complications and ileocolonic procedures. As patients were not identified personally, multiple admissions per year cannot be excluded. Furthermore, large hospital discharge data-based cohort studies are potentially biased by possible mislabeling and inaccurate coding behavior, which cannot be excluded nor controlled for. Additionally, for some years, exact numbers cannot be presented due to confidentiality reasons. The latter occurs when too little overall case numbers might allow tracing of individual patients. In particular, regarding postoperative complications, only 2010, 2012, 2015, and 2016 were available for our analysis. Further, the postoperative complication “anastomotic leakage” has been introduced in 2013 so data extraction was based on 2015 and 2016. Hence, for some of the complications, data has been pooled to allow for overall estimation of current developments. However, given these limitations, the predominant advantage of this study is the large data set. While other studies represent only a fraction of all cases, our study included all inpatient cases of Crohn’s disease admitted to the hospital in Germany. This corresponds to 201,165 patients over the entire study period.

The results indicate that despite an increase in conservative therapeutic options, the rate of surgical treatment of Crohn’s disease in German hospitals remained stable in recent years. In addition, both the mortality rate and the rate of complications were low. All in all, it can therefore be concluded that surgery as a therapeutic option for Crohn’s disease has its own significance. Recent studies have shown that both drug therapy and surgical options should be considered as equal therapeutic strategies, which is why current guidelines mandate interdisciplinary consultation prior to intensifying treatment options [18–21]. While in some cases, the use of biologicals leads to remission of the disease, in selected subgroups of patients, early surgery may be associated with good surgical outcomes [8, 22]. In particular, laparoscopic ileocolonic resection might be an attractive alternative to several years of drug therapy and can lead to long-lasting remission [23]. For these reasons, it is critical that surgical management of CD patients be individualized in the biological era, and case-based, multidisciplinary decision-making is crucial to achieve favorable outcomes.

## Supplementary Information

ESM 1 (DOCX 21 kb)

## Data Availability

Data can be retrieved from the national diagnosis-related groups (DRG) statistics which contains all German data records of inpatient treatments billed according to the DRG system in Germany.
