# Supplementary material for: Crohn’s disease: a population-based study of surgery in the age of biological therapy
Source: Int J Colorectal Dis. 2021 Apr 19;36(11):2419–26. doi: 10.1007/s00384-021-03930-w (PMC8505365; doi:10.1007/s00384-021-03930-w)
Supplement: Supplementary file 1 — (DOCX 21 kb) [file 384_2021_3930_MOESM1_ESM.docx]

**Supplemental Data**

**Supplemental Table 1 Definition of patient population and procedure codes (OPS)**

|  | **ICD and OPS codes** |
| --- | --- |
| **Patient population** |  |
| All inpatient cases with Crohn´s disease | K50.0, K50.1, K50.8-, K50.9 |
| **Secondary diagnoses** |  |
| Intestinal perforation | ICD-10 K63.1 |
| Intraabdominal abscess | ICD-10 K63.0 |
| Intestinal fistula | ICD-10 K63.2 |
| Peritonitis | ICD-10 K65.- |
| Stenosis or stricture of the intestine | ICD-10 K56.6 |
| Intestinal obstruction (Ileus, unspecified) | ICD-10 K56.7 |
| Intestinal bleeding | ICD-10 K92.2 |
| Mild malnutrition | ICD-10 E44.1 |
| Moderate malnutrition | ICD-10 E44.0 |
| Severe malnutrition | ICD-10 E43 |
| **Surgical approach** |  |
| Open ileocaecal resection | OPS 5-455.21 |
| Laparoscopic ileocaecal resection | OPS 5-455.25 |
| Converted lap. ileocaecal resection | OPS 5-455.27 |
| Open right-sided hemicolectomy | OPS 5-455.41 |
| Laparoscopic right-sided hemicolectomy | OPS 5-455.45 |
| Converted lap. right-sided hemicolectomy | OPS 5-455.47 |
| **Postoperative complications** |  |
| Anastomotic leakage | ICD-10 K91.83^1^ |
| Intestinal obstruction | ICD-10 K91.3 |
| Wound dehiscence | ICD-10 T81.3 |
| Surgical site infection | ICD-10 T81.4 |

*^1^Available from 2013*

*OPS: Procedure code (“Operationen- und Prozedurenschlüssel“)*
